# Exploratory, Randomized, Dose-Response Study of the Anti-PD-L1 Antibody HFC-L1/c4G12 in Dogs with Pulmonary Metastatic Oral Malignant Melanoma

**DOI:** 10.3390/vetsci12090850

**Published:** 2025-09-02

**Authors:** Kenji Hosoya, Sangho Kim, Ryohei Kinoshita, Naoya Maekawa, Satoru Konnai, Satoshi Takagi, Michihito Tagawa, Yumiko Kagawa, Tatsuya Deguchi, Ryo Owaki, Yurika Tachibana, Madoka Yokokawa, Hiroto Takeuchi, Hayato Nakamura, Akinori Yamauchi, Ayano Kudo, Shintaro Kamo, Yukinari Kato, Shigeki Kanazawa, Tomoyuki Abe, Takuya Furuta, Keiichi Yamamoto, Yasuhiko Suzuki, Tomohiro Okagawa, Shiro Murata, Kazuhiko Ohashi

**Affiliations:** 1 Veterinary Teaching Hospital, Faculty of Veterinary Medicine, Hokkaido University, Sapporo 060-0819, Japan; 2 Cancer Research Unit, One Health Research Center, Hokkaido University, Sapporo 060-0818, Japan; 3 Department of Advanced Pharmaceutics, Faculty of Veterinary Medicine, Hokkaido University, Sapporo 060-0818, Japan; 4 Department of Disease Control, Faculty of Veterinary Medicine, Hokkaido University, Sapporo 060-0818, Japan; 5 Institute for Vaccine Research and Development (HU-IVReD), Hokkaido University, Sapporo 001-0021, Japan; 6 Veterinary Research Unit, International Institute for Zoonosis Control, Hokkaido University, Sapporo 001-0020, Japan; 7 Laboratory of Small Animal Surgery, School of Veterinary Medicine, Azabu University, Sagamihara 252-5201, Japan; 8 Veterinary Medical Center, Obihiro University of Agriculture and Veterinary Medicine, Obihiro 080-8555, Japan; 9 North Lab, Sapporo 003-0027, Japan; 10 Companion Animal Internal Medicine, Department of Companion Animal Clinical Sciences, School of Veterinary Medicine, Rakuno Gakuen University, Ebetsu 069-8501, Japan; 11 Department of Antibody Drug Development, Tohoku University Graduate School of Medicine, Sendai 980-8575, Japan; 12 FUSO Pharmaceutical Industries, Ltd., Osaka 541-0045, Japan; 13 Division of Bioresources, International Institute for Zoonosis Control, Hokkaido University, Sapporo 001-0020, Japan; 14 Global Station for Zoonosis Control, Global Institution for Collaborative Research and Education (GI-CoRE), Hokkaido University, Sapporo 060-0808, Japan; 15 International Affairs Office, Faculty of Veterinary Medicine, Hokkaido University, Sapporo 060-0818, Japan

**Keywords:** canine tumor, immune checkpoint inhibitor, immunotherapy, malignant melanoma, programmed death ligand 1 (PD-L1)

## Abstract

Oral malignant melanoma (OMM) is a highly aggressive malignancy in dogs. The development of effective systemic therapies is urgently required to improve the treatment of canine OMM. Immunotherapy using immune checkpoint inhibitors (ICIs) has been investigated in canines following their dramatic success in human cancer treatment; however, there is still a need for extensive veterinary clinical studies to clarify and optimize their clinical benefits. Among the ICIs under development for canine cancer immunotherapy, c4G12 (HFC-L1), a canine chimeric anti-PD-L1 antibody, has shown promising efficacy in dogs with pulmonary metastatic OMM in previous clinical studies. However, the optimal dose of HFC-L1/c4G12 has not yet been determined. To explore the dose–response relationship of HFC-L1, a multicenter, randomized clinical study was conducted using three different doses (2, 5, or 10 mg/kg via intravenous infusion every 2 weeks) to treat dogs with pulmonary metastatic OMM (*n* = 8–9 per group). The safety profiles were similar among the dose groups, and numerically longer median overall survival was achieved in the higher dose groups (5 and 10 mg/kg) than in the 2 mg/kg group. Although the study was exploratory in nature with a small sample size, 5–10 mg/kg should be considered the preferred dose in future clinical studies using HFC-L1.

## Simple Summary

Pulmonary metastatic oral malignant melanoma is a highly aggressive cancer in dogs, and effective systemic therapies are urgently needed. Immune checkpoint inhibitors, which help the immune system attack cancer cells, have shown promise in canine studies. This exploratory, randomized clinical study evaluated the safety and efficacy of an anti-PD-L1 antibody, HFC-L1 (also known as c4G12), in dogs with pulmonary metastatic oral malignant melanoma. Twenty-six dogs were treated with three different doses of HFC-L1 (2, 5, or 10 mg/kg every 2 weeks). The safety profiles were similar across all dose groups, and no severe treatment-related adverse events were observed. Dogs treated with higher doses (5 or 10 mg/kg) showed numerically longer overall survival compared to the 2 mg/kg group. These findings suggest that HFC-L1 therapy is well tolerated and that higher doses may provide improved clinical benefit. Although the study was exploratory in nature with a small sample size, the results support the use of 5–10 mg/kg as the preferred dose in future clinical studies. This research contributes to the development of immunotherapy for canine cancers and may also inform comparative oncology approaches relevant to human medicine.

## 1. Introduction

Malignant melanoma is a relatively common neoplasm in dogs that often arises in the haired skin, digits (nail bed), and oral cavity [1,2]. Oral malignant melanoma (OMM) is the most common oral malignancy in dogs, with diverse but frequently aggressive biological behaviors [1]. The most effective local treatment for OMM is surgery, with early diagnosis and early intervention being associated with successful outcomes [3,4]. Radiation therapy also plays an important role in the local control of OMM, with reported response rates typically exceeding 80% [5,6,7,8]. However, OMM is often highly invasive and metastatic; thus, for high stages of OMM, the development of systemic therapies is urgently required. Conventional (cytotoxic) chemotherapy does not appear to correlate with effective OMM management, with only modest response rates reported in the literature [9,10,11,12]. The lungs are the most common site of distant metastasis in OMM, and there is no effective systemic treatment available for pulmonary metastatic OMM that is capable of improving survival of affected dogs.

Immunotherapy has been proposed as an effective systemic therapy for malignant melanoma in humans [13]. Among these, immune checkpoint inhibitors (ICIs), such as anti-cytotoxic T lymphocyte-associated protein 4 (CTLA-4), anti-programmed death 1 (PD-1), and anti-PD ligand 1 (PD-L1) antibodies, have been reported to improve overall survival (OS) in patients with advanced disease [14,15,16] and have become standard therapies in the past decade. Similar research efforts have been made in dogs, and previous studies have revealed that tumor-infiltrating lymphocytes collected from OMM express the immunosuppressive receptor PD-1 on their surface, and PD-L1, a ligand for PD-1, is expressed in the tumor tissues of OMM at a high rate (73–100%) [17,18,19,20]. The blockade of the PD-1/PD-L1 axis by monoclonal antibodies improved cytokine production and lymphocyte proliferation in canine immune cell cultures [17,21,22], strongly suggesting that clinical intervention with anti-PD-1 or anti-PD-L1 antibody drugs could exert antitumor effects in dogs with OMM by reinvigorating T cell-mediated immunity against the tumor. Indeed, early pilot clinical studies have shown promising antitumor efficacy of these ICIs in canine OMM [18,21,22]; for example, an anti-PD-L1 antibody, c4G12, induced an objective response in 7.7–14.3% of dogs with OMM, and a survival benefit was strongly suggested in comparison to a historical control group treated with standard therapies at the same veterinary hospital [18,22]. These preliminary studies have encouraged further development of ICIs for canine OMM.

In previous clinical studies using c4G12, the antibody drug was administered at 5 mg/kg every 2 weeks in most cases, with some exceptions in which dogs were treated at 2 mg/kg, based on the veterinarians’ discretion [18,22,23,24]. However, the optimal dose of c4G12 for both safety and clinical efficacy has not yet been determined. In this study, to explore the dose–response relationship of c4G12 (renamed here as HFC-L1), we conducted a multicenter, randomized clinical study involving 26 dogs, using three different doses of HFC-L1: 2, 5, and 10 mg/kg.

## 2. Materials and Methods

### 2.1. Overview of the Clinical Study

This clinical study was designed as a multicenter, randomized, non-blinded, dose–response study of HFC-L1 to explore the optimal dosage for treating canine pulmonary metastatic OMM. The clinical study was conducted at the veterinary teaching hospitals of Hokkaido University (HU), Azabu University (AU), and Obihiro University of Agriculture and Veterinary Medicine (OUAVM) between November 2021 and February 2024, with the approval of the Ethics Committee or Institutional Animal Care Committee (approval numbers: HU, 2021-08; AU, 19403-5 and 220308-1; OUAVM, 21-164). Dogs met the following inclusion criteria for enrolment in the clinical study: (1) dogs diagnosed with stage IV OMM, as defined by the TNM-based World Health Organization staging scheme [25], and with pulmonary metastasis (PM); and (2) dogs for whom written informed consent was obtained from the owners. Dogs that met at least one of the following exclusion criteria were excluded from the study: (1) dogs with severe systemic illnesses unrelated to the tumor; (2) dogs that had experienced severe immune-related disorders that might recur during the study; (3) dogs with difficulty in hospital revisits and follow-up observation because of planned relocation or hospital transfer; (4) dogs with any difficulty adhering to the scheduled revisit for drug administration and clinical examinations; (5) dogs that were (potentially) pregnant or lactating; (6) dogs that had received another experimental therapy within 12 weeks prior to enrolment or were participating in another clinical study at the time of enrolment; or (7) dogs considered by the investigators to be unsuitable for participation in the clinical study. HFC-L1 was prepared as a 10 mg/kg antibody solution in phosphate-buffered saline (FUSO Pharmaceutical Industries, Ltd., Osaka, Japan) and stored below −20 °C until use. Dogs with OMM (with PM, *n* = 26) were randomly assigned to three dose groups: 2 mg/kg (*n* = 9), 5 mg/kg (*n* = 9), and 10 mg/kg (*n* = 8). The doses were selected based on our previous clinical studies [18,22,23,24] and a preliminary safety assessment conducted in a healthy laboratory dog, in which a single administration of 10 mg/kg c4G12 was well tolerated. Treatment duration was set to 24 weeks (for safety and response evaluation), which could be extended upon reasonable request from the owner for up to 48 weeks for additional safety assessment. When formalin-fixed, paraffin-embedded tumor tissues (biopsied at any time point) were available, PD-L1 expression in tumor cells was evaluated by immunohistochemistry at a commercial pathology laboratory (North Lab, Sapporo, Japan), as described previously [18]. HFC-L1 was diluted in saline and administered intravenously over 1 h using a syringe pump every 2 weeks. Prior to administration, the use of antihistamine drugs was allowed as premedication.

### 2.2. Assessment of Safety

Routine follow-up, including physical examination, complete blood count, and blood chemistry, was performed every 2 weeks to monitor adverse events. After the first 6 weeks, blood tests were scheduled every 6 weeks. Additional assessments for adverse events [including urinalysis, thoracic or abdominal radiography, ultrasonography, computed tomography (CT), and magnetic resonance imaging] were performed when clinically required. Adverse events were classified and graded according to the Veterinary Cooperative Oncology Group–Common Terminology Criteria for Adverse Events (VCOG-CTCAE) v1.1 [26]. The attribution (causality) of adverse events was categorized as related, unrelated, or indeterminate by the veterinary clinicians. Adverse events possibly related to HFC-L1 therapy (related or indeterminate) were considered treatment-related adverse events (TRAEs). Pearson’s chi-squared test was used to compare the frequency of TRAEs (of any grade) between dose groups.

### 2.3. Evaluation of Tumor Response

Tumor response to HFC-L1 treatment was defined according to the response evaluation criteria for solid tumors in dogs (cRECIST) v1.0 [27]. Tumor size was routinely evaluated by clinical examination, thoracic radiography, or CT every 6 weeks, using the same modality as the baseline assessment. Dogs with measurable, target lesion(s) (i.e., ≥10 mm on clinical examination or CT; ≥20 mm on thoracic radiograph) at baseline were considered “with target disease” (*n* = 4) and subjected to response evaluation. The remaining 22 dogs had only non-measurable lesions at baseline and were thus excluded from the response evaluation. Tumor response was defined as: complete response (CR) if all detectable tumors disappeared in response to the treatment, partial response (PR) if the tumor burden was reduced by ≥30%, progressive disease (PD) if the tumor burden increased by ≥20% or new lesion(s) appeared, and stable disease (SD) if the tumor burden remained unchanged (decreased by <30% or increased by <20%) for at least 6 weeks. When re-evaluation of the tumor burden could not be performed for any reason, the tumor response was reported as not evaluable (NE).

### 2.4. Evaluation of Survival

The OS of the dogs was defined as the time (days) from the first HFC-L1 dose to death. The survival after the diagnosis of PM was defined as the time (days) from the first diagnosis of PM to death [18,22]. Dogs that were lost to follow-up or still alive at the end of the observation period (February 2024) were included in the survival analysis as censored data. Kaplan–Meier curves were generated, and statistical analysis was performed using the log-rank test.

Dogs that survived for >4 months on HFC-L1 treatment were considered long-term survivors, based on a previous finding that dogs with pulmonary metastatic OMM treated with the best available therapy and/or supportive care (historical control group, *n* = 15) typically died within 4 months, with a median survival after the diagnosis of PM of 54 days (range: 7–111 days) [22].

### 2.5. Statistical Analysis

All statistical analyses were performed using EZR (version 1.35) [28], and *p* < 0.05 was considered statistically significant.

## 3. Results

### 3.1. Baseline Characteristics of the Study Population

Twenty-six dogs were enrolled, including 18 dogs at HU, seven dogs at AU, and one dog at OUAVM. All dogs were histopathologically or cytopathologically diagnosed with malignant melanoma originating from the oral cavity, and clinical evidence of PM was confirmed by diagnostic imaging using chest radiography or CT. The dogs were randomly assigned to three dose groups: 2 mg/kg (*n* = 9), 5 mg/kg (*n* = 9), and 10 mg/kg (*n* = 8). Various canine breeds were included, with miniature dachshunds (*n* = 8) and toy poodles (*n* = 4) being the most common across all dose groups. The median ages at the time of enrolment were 12, 12, and 15 years for the 2 mg/kg, 5 mg/kg, and 10 mg/kg groups, respectively. PD-L1 expression was evaluated in archived tumor tissue samples subjected to histopathological diagnosis from 17 dogs, and all samples, except for one in the 2 mg/kg group, were PD-L1–positive. Because most samples were PD-L1–positive, subpopulation analyses based on PD-L1 status were not performed in this study. Measurable lesions, as defined by cRECIST [27], were present in two dogs in the 2 mg/kg group, one dog in the 5 mg/kg group, and one dog in the 10 mg/kg group; thus, these dogs (*n* = 4) were eligible for response evaluation (Table 1).

### 3.2. Safety of HFC-L1 Therapy

All TRAEs are listed in Table 2. TRAEs of any grade were reported in three (33.3%), five (55.6%), and three (37.5%) dogs in the 2, 5, and 10 mg/kg groups, respectively. The frequency of TRAEs was not significantly different between groups (*p* = 0.601). Grade 3 TRAEs were observed in three dogs: anaphylaxis (2 mg/kg group), elevated blood urea nitrogen (BUN) (5 mg/kg group), and anorexia and weight loss (10 mg/kg group). No grade 4 or 5 TRAEs were observed. Common TRAEs included elevated alanine aminotransferase (ALT), elevated creatinine, elevated BUN, anorexia, diarrhea, and vomiting. Two dogs discontinued HFC-L1 therapy because of TRAEs: elevated BUN and creatinine (both grade 2) in one dog (5 mg/kg group), and anaphylaxis (grade 3) in another dog (2 mg/kg group).

### 3.3. Tumor Response to HFC-L1 Therapy

**Figure 1:**
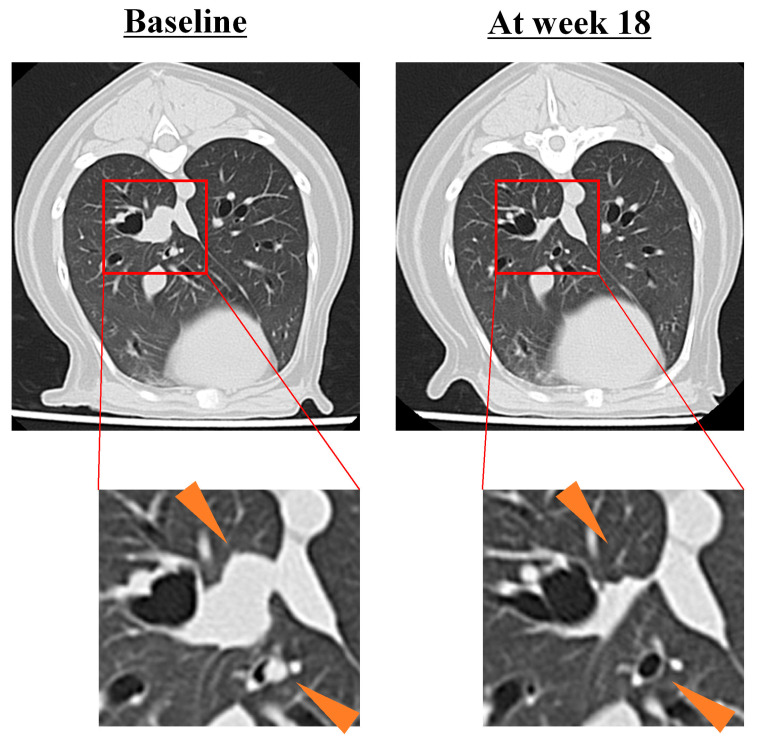
Antitumor efficacy of HFC-L1. A measurable pulmonary metastatic lesion of oral malignant melanoma (center, 13 mm at baseline) responded to 5 mg/kg HFC-L1 therapy at week 18 (6 mm, partial response). A non-measurable lesion (lower right) at baseline was undetectable at week 18.

To achieve local tumor control, most dogs had received prior surgery and/or radiation therapy for the primary tumor before enrolment in this clinical study; thus, measurable target lesions were not available for these dogs and were excluded from the response evaluation. Among dogs with target disease (*n* = 4), one dog (5 mg/kg group) experienced PR at week 18 of HFC-L1 treatment (Figure 1), with the longest diameter of the target lesion in the lung reduced by 54% (from 13 mm at baseline to 6 mm at week 18) and disappearance of some non-measurable (non-target) lesions. Another dog (2 mg/kg group) experienced PD as its best overall response, and the other two dogs either died (2 mg/kg group) or dropped out (10 mg/kg group) before the first tumor evaluation (NE, Table 3).

### 3.4. Comparison of OS

**Figure 2:**
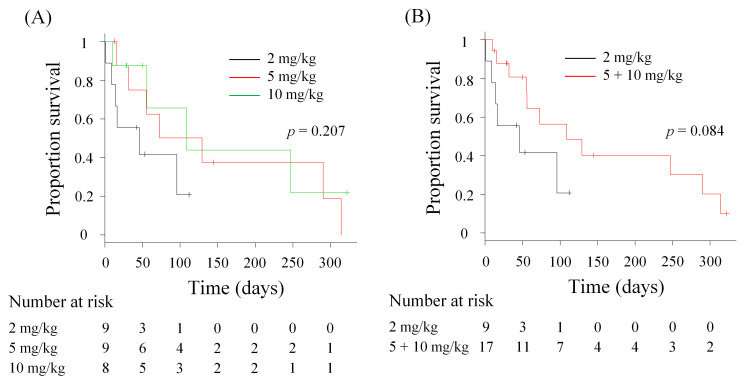
Overall survival (OS) of dogs treated with HFC-L1. (**A**) Comparison of OS among the three dose groups (*n* = 8–9). (**B**) Comparison of OS between the 2 mg/kg group (*n* = 9) and higher-dose group (5 mg/kg and 10 mg/kg, *n* = 17). Marks on the line indicate censored data. Statistical analysis was performed using the log-rank test.

The median OS of HFC-L1–treated dogs was 46 days [95% confidence interval (CI): 1–not applicable (NA) days] in the 2 mg/kg group, 101 days (95% CI: 15–315 days) in the 5 mg/kg group, and 109 days (95% CI: 10–NA days) in the 10 mg/kg group. The median OS was numerically longer in the 5 and 10 mg/kg groups than that in the 2 mg/kg group; however, there was no significant difference between the groups (*p* = 0.207, Figure 2A). When the higher-dose groups (5 and 10 mg/kg) were combined for further analysis, the median OS in the combined group was 109 days (*n* = 17, 95% CI: 55–290 days). Again, no significant difference was found between the combined and 2 mg/kg groups (*p* = 0.084, Figure 2B). To further assess the survival benefit of HFC-L1 therapy, we defined dogs with an OS > 4 months as long-term survivors. In the 2 mg/kg, 5 mg/kg, and 10 mg/kg groups, 0% (0/9, 95% CI: 0–28.3%), 44.4% (4/9, 95% CI: 13.7–78.8%), and 25.0% (2/8, 95% CI: 3.2–65.1%) of dogs, respectively, were considered long-term survivors (Supplementary Table S1).

### 3.5. Comparison of Survival After the Diagnosis of PM

To enable comparisons with the historical control data, survival after the diagnosis of PM was calculated for dogs treated with HFC-L1 at HU. Since the historical control data were collected at HU [18,22], dogs treated at AU or OUAVM were excluded from the analyses. The median survival after the diagnosis of PM for the historical control (*n* = 15), 2 mg/kg (*n* = 6), 5 mg/kg (*n* = 6), and 10 mg/kg (*n* = 6) groups were 54 days (95% CI: 25–NA days), 82 days (95% CI: 44–NA days), 133.5 days (95% CI: 29–NA days), and 259 days (95% CI: 66–NA days), respectively. The survival was significantly longer in the 10 mg/kg group (*p* = 0.032), whereas no significant difference was observed in the 2 mg/kg (*p* = 0.363) and 5 mg/kg (*p* = 0.105) groups (Supplementary Figure S1A–C). The survival was significantly longer in the combined, higher-dose group (5 mg/kg plus 10 mg/kg, *n* = 12, *p* = 0.015) when compared to the historical control (Supplementary Figure S1D), with the median survival after the diagnosis of PM of 156 days (95% CI: 46–326 days).

Together, these findings suggest that the clinical benefit of HFC-L1 treatment is more apparent in the higher-dose groups.

## 4. Discussion

In this exploratory, randomized, dose–response study of HFC-L1, the type, frequency, and severity of TRAEs were similar between the dose groups, suggesting that HFC-L1 therapy was well tolerated at doses up to 10 mg/kg every 2 weeks. The OS of the treated dogs did not significantly differ among the groups; however, a numerically longer median OS was reported in the 5 and 10 mg/kg groups than in the 2 mg/kg group. Collectively, these findings suggest that HFC-L1 therapy at doses of 5 or 10 mg/kg may provide improved survival without increasing toxicity.

The major limitation of the current clinical study was the small sample size for each dose group (*n* = 8–9), which made it difficult to find statistically significant differences between the groups. Given that a clinically significant difference in OS has been suggested, the potential risk of a type II error should be taken into account when interpreting the survival data. In addition, tumor response was evaluable in only four dogs, rendering comparison of response rates between dose groups infeasible; thus, treatment efficacy was compared across dose groups using survival data alone. Moreover, the clinical benefit of the 2 mg/kg group was ambiguous because of the absence of a placebo control (0 mg/kg) group, which was omitted for ethical reasons. In previous studies, some dogs that received 2 mg/kg c4G12 therapy showed objective responses [22,24], and it is expected that the 2 mg/kg group in this study would have clinical benefits to a certain, if not the highest, extent. However, the survival benefit seemed suboptimal in the 2 mg/kg group, given its numerically shorter median OS and the absence of long-term survivors in this group. Therefore, to reduce the risk of underdosing, the higher dose is recommended for the future development of HFC-L1, provided that it is not associated with an increased incidence of side effects, including immune-related toxicities.

The TRAEs reported in this study were consistent with those of previous reports on c4G12 in dogs [18,22,23,24], and no novel safety concerns were identified. The overall safety profiles were similar between the dose groups, supporting the use of high doses (5–10 mg/kg) for the future development of HFC-L1. Potential immune-related adverse events (irAEs) observed in this study included vomiting/diarrhea, elevated liver enzymes, and increased BUN/creatinine levels, which were suggestive of gastrointestinal, hepatic, and renal toxicities, respectively. Careful monitoring of these organs is needed in future studies using HFC-L1. In human clinical studies using ICIs, other types of irAEs, including cutaneous, endocrine, pulmonary, and nervous system toxicities have been reported [29,30], all of which may also occur in dogs. The frequency of TRAEs that led to treatment discontinuation was considerably low in this study [11.1% (1/9), 11.1% (1/9), and 0% (0/8) in the 2, 5, and 10 mg/kg groups, respectively], and the safety profiles of HFC-L1 at the tested doses were considered acceptable, given the highly aggressive nature of pulmonary metastatic OMM.

In conclusion, although the study was exploratory in nature with a small sample size, this multicenter, randomized, dose–response study of HFC-L1 demonstrated that a high dose (5 or 10 mg/kg) was associated with a numerically longer OS and acceptable safety profiles. We propose that 5–10 mg/kg is the preferred dose in future clinical studies using HFC-L1/c4G12. As future directions, a large-scale confirmatory study for optimal dose determination should be conducted with appropriate stratification factors (e.g., PD-L1 status and prior therapies). In addition, to maximize the clinical benefit of HFC-L1/c4G12 therapy, biomarkers for predicting treatment outcomes and combination strategies to enhance therapeutic efficacy (e.g., with radiation [23] or other immune modulators [31]) should be investigated in subsequent clinical studies.

## Figures and Tables

**Table 1:** Characteristics of dogs at baseline of HFC-L1 therapy.

	2 mg/kg (*n* = 9)	5 mg/kg (*n* = 9)	10 mg/kg (*n* = 8)
Breed―No. (%)			
American Cocker Spaniel	0	1 (11.1)	0
Chihuahua	0	1 (11.1)	1 (12.5)
Miniature Dachshund	4 (44.4)	1 (11.1)	3 (37.5)
Miniature Schnauzer	1 (11.1)	0	0
Norfolk Terrier	0	1 (11.1)	0
Shetland Sheepdog	0	1 (11.1)	0
Shiba	0	1 (11.1)	0
Tosa	1 (11.1)	0	0
Toy Poodle	1 (11.1)	1 (11.1)	2 (25.0)
Welsh Corgi	0	0	1 (12.5)
Yorkshire Terrier	0	0	1 (12.5)
Mix	2 (22.2)	2 (22.2)	0
Age―year			
Median	12	12	15
Range	6–16	9–15	13–19
Sex―No. (%)			
Male, intact	2 (22.2)	0	2 (25.0)
Male, castrated	0	5 (55.6)	3 (37.5)
Female, intact	1 (11.1)	0	0
Female, spayed	6 (66.7)	4 (44.4)	3 (37.5)
PD-L1 expression―No. (%)			
Positive	5 (55.6)	5 (55.6)	6 (75.0)
Negative	1 (11.1)	0	0
Not determined	3 (33.3)	4 (44.4)	2 (25.0)
Measurable lesion(s)―No. (%)			
Present	2 (22.2)	1 (11.1)	1 (12.5)
Absent	7 (77.8)	8 (88.9)	7 (87.5)

PD-L1, programmed death-ligand 1.

**Table 2:** Treatment-related adverse events (TRAEs).

TRAEs―No. (%)	2 mg/kg (*n* = 9)		5 mg/kg (*n* = 9)		10 mg/kg (*n* = 8)	
	Any Grade	Grade 3	Any Grade	Grade 3	Any Grade	Grade 3
Any TRAEs	3 (33.3)	1 (11.1)	5 (55.6)	1 (11.1)	3 (37.5)	1 (12.5)
ALP, high	0	0	0	0	1 (12.5)	0
ALT, high	1 (11.1)	0	0	0	1 (12.5)	0
AST, high	1 (11.1)	0	0	0	0	0
Creatinine, high	0	0	2 (22.2)	0	2 (25.0)	0
BUN, high	0	0	2 (22.2)	1 (11.1)	0	0
Anorexia	1 (11.1)	0	0	0	1 (12.5)	1 (12.5)
Allergic reaction	0	0	0	0	1 (12.5)	0
Anaphylaxis	1 (11.1)	1 (11.1)	0	0	0	0
Diarrhea	1 (11.1)	0	2 (22.2)	0	1 (12.5)	0
Vomiting	1 (11.1)	0	3 (33.3)	0	0	0
Weight loss	0	0	0	0	1 (12.5)	1 (12.5)

ALP, alkaline phosphatase; ALT, alanine aminotransferase; AST, aspartate aminotransferase; BUN, blood urea nitrogen.

**Table 3:** Tumor response to HFC-L1 therapy in dogs with target disease.

Best Overall Response―No. (%)	2 mg/kg (*n* = 2)	5 mg/kg (*n* = 1)	10 mg/kg (*n* = 1)
CR	0	0	0
PR	0	1 (100.0)	0
SD	0	0	0
PD	1 (50.0)	0	0
NE	1 (50.0)	0	1 (100.0)

CR, complete response; PR, partial response; SD, stable disease; PD, progressive disease; NE, not evaluable.

## Data Availability

The original contributions presented in this study are included in the article/Supplementary Material. Further inquiries can be directed to the corresponding authors.

## Supplementary Materials

The following supporting information can be downloaded at: https://www.mdpi.com/article/10.3390/vetsci12090850/s1, Supplementary Figure S1: Survival after the diagnosis of pulmonary metastasis (PM) in dogs treated with HFC-L1. Supplementary Table S1: Characteristics of dogs treated with HFC-L1 therapy.

## Abbreviations

The following abbreviations are used in this manuscript:

AU	Azabu University
BUN	Blood Urea Nitrogen
CR	Complete Response
CT	Computed Tomography
CI	Confidence Interval
HU	Hokkaido University
ICI	Immune Checkpoint Inhibitor
irAE	Immune-Related Adverse Event
NE	Not Evaluable
OUAVM	Obihiro University of Agriculture and Veterinary Medicine
OMM	Oral Malignant Melanoma
OS	Overall Survival
PR	Partial Response
PD-1	Programmed Death 1
PD-L1	Programmed Death Ligand 1
PD	Progressive Disease
PM	Pulmonary Metastasis
cRECIST	Response Evaluation Criteria for Solid Tumours in Dogs
SD	Stable Disease
TRAE	Treatment-Related Adverse Event
VCOG-CTCAE	Veterinary Cooperative Oncology Group–Common Terminology Criteria for Adverse Events
