# Supplementary material for: Exploratory, Randomized, Dose-Response Study of the Anti-PD-L1 Antibody HFC-L1/c4G12 in Dogs with Pulmonary Metastatic Oral Malignant Melanoma
Source: Vet Sci. 2025 Sep 2;12(9):850. doi: 10.3390/vetsci12090850 (PMC12474000; doi:10.3390/vetsci12090850)
Supplement: Supplementary file 1 [file vetsci-12-00850-s001.zip › vetsci-3814375-supplementary.pdf]

Supplementary Materials for:

Exploratory, randomized, dose-response study of the anti-PD-L1 antibody HFC-L1/c4G12 in dogs with pulmonary metastatic oral malignant melanoma

Kenji Hosoya†, Sangho Kim†, Ryohei Kinoshita†, Naoya Maekawa†, Satoru Konnai*, Satoshi Takagi, Michihito Tagawa, Yumiko Kagawa, Tatsuya Deguchi, Ryo Owaki, Yurika Tachibana, Madoka Yokokawa, Hiroto Takeuchi, Hayato Nakamura, Akinori Yamauchi, Ayano Kudo, Shintaro Kamo, Yukinari Kato**, Shigeki Kanazawa, Tomoyuki Abe, Takuya Furuta, Keiichi Yamamoto, Yasuhiko Suzuki, Tomohiro Okagawa, Shiro Murata, Kazuhiko Ohashi.

†These authors contributed equally to this work.

*Corresponding author (konnai@vetmed.hokudai.ac.jp)
**Co-corresponding author (yukinari.kato.e6@tohoku.ac.jp)

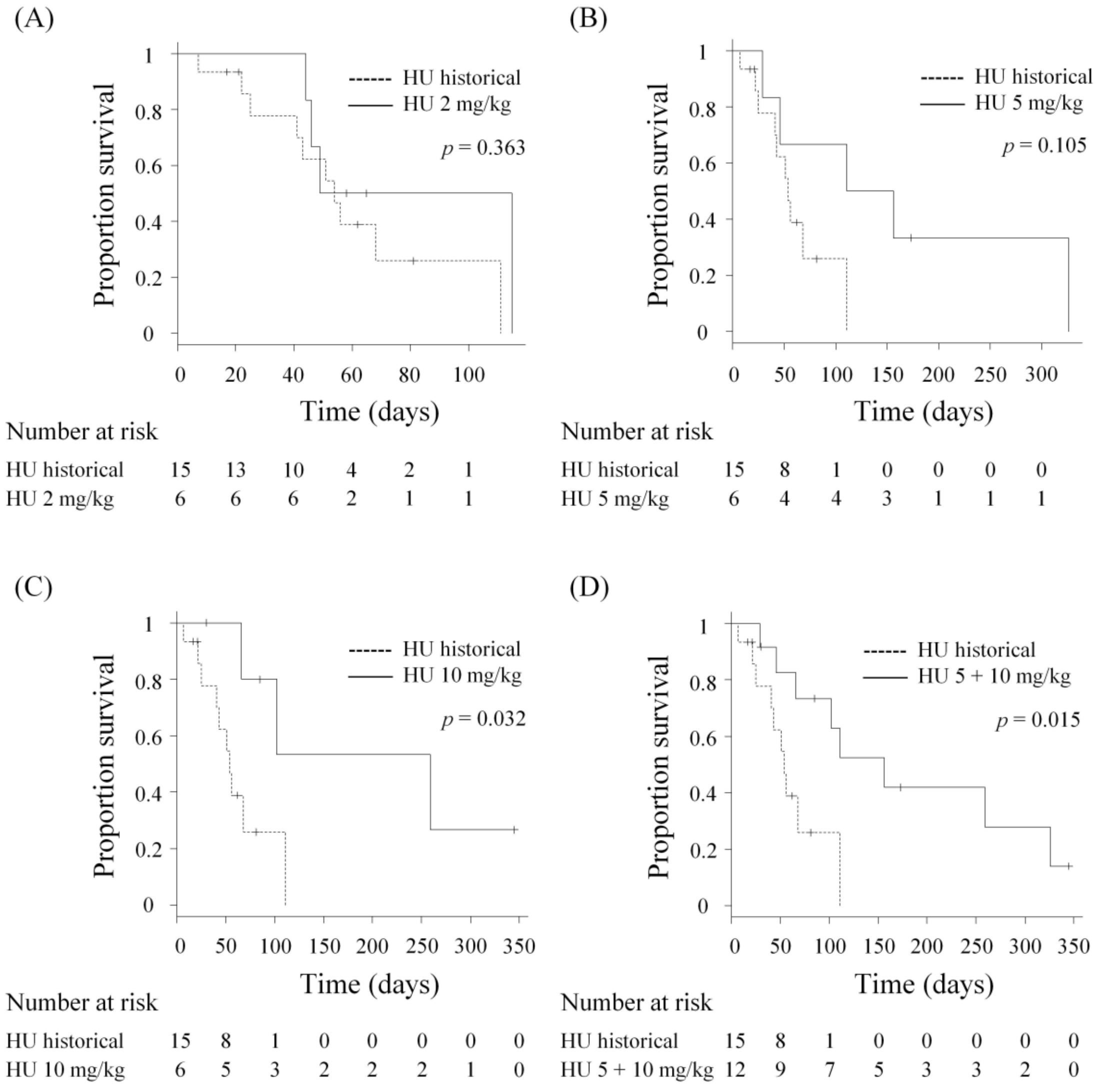

Supplementary Figure S1. Survival after the diagnosis of pulmonary metastasis (PM) in dogs treated with HFC-L1.

The survival from the first diagnosis of PM to death was compared with the historical control group. Comparison of the survival between the (A) 2 mg/kg ($n = 6$), (B) 5 mg/kg ($n = 6$), (C) 10 mg/kg ($n = 6$), or higher dose (5 mg/kg and 10 mg/kg, $n = 12$) group and the historical control group ($n = 15$). Marks on the line indicate censored data. Statistical analysis was performed using the log-rank test.

Supplementary Table S1. Characteristics of dogs treated with HFC-L1 therapy.

Dog #	Institution	Dose (mg/kg)	Breed	Age (years)	Sex	PD-L1 expression	Target lesion	BOR	OS (days)
1	HU	5	American Cocker Spaniel	14	Female, spayed	+	Absent	NA	314
2	HU	2	Mix	16	Female, spayed	ND	Absent	NA	96
3	HU	10	Miniature Dachshund	19	Male	+	Absent	NA	50*
4	HU	5	Miniature Dachshund	15	Female, spayed	+	Absent	NA	129
5	HU	2	Tosa	6	Female, spayed	ND	Absent	NA	42*
6	HU	10	Chihuahua	17	Female, spayed	+	Absent	NA	247
7	HU	2	Toy Poodle	12	Female, spayed	+	Absent	NA	9
8	HU	10	Miniature Dachshund	15	Male, castrated	ND	Absent	NA	56
9	HU	5	Chihuahua	12	Female, spayed	+	Absent	NA	15
10	HU	10	Toy Poodle	13	Female, spayed	+	Absent	NA	10
11	HU	2	Mix	12	Female, spayed	+	Absent	NA	16
12	HU	5	Mix	9	Male, castrated	+	Absent	NA	55
13	HU	10	Yorkshire Terrier	13	Male	+	Absent	NA	28*
14	HU	2	Miniature Dachshund	11	Female, spayed	+	Absent	NA	53*
15	HU	10	Welsh Corgi	15	Male, castrated	+	Absent	NA	322*
16	HU	5	Shiba	13	Female, spayed	ND	Absent	NA	32
17	HU	5	Shetland Sheepdog	15	Male, castrated	+	Absent	NA	145*
18	HU	2	Miniature Dachshund	13	Female, spayed	+	Absent	NA	14
19	AU	2	Miniature Schnauzer	11	Male	ND	Present	NE	46
20	AU	10	Miniature Dachshund	16	Male, castrated	+	Present	NE	29*
21	AU	5	Toy Poodle	12	Male, castrated	ND	Present	PR	290
22	AU	5	Mix	9	Male, castrated	ND	Absent	NA	73

23	AU	10	Toy Poodle	13	Female, spayed	ND	Absent	NA	109
24	AU	2	Miniature Dachshund	15	Female	−	Absent	NA	1
25	AU	5	Norfolk Terrier	11	Male, castrated	ND	Absent	NA	13*
26	OUAVM	2	Miniature Dachshund	11	Male	+	Present	PD	112*

HU, Hokkaido University; AU, Azabu University; OUAVM, Obihiro University of Agriculture and Veterinary Medicine.

BOR, best overall response; OS, overall survival; ND, not determined; NA, not applicable; NE, not evaluable; PR, partial response; PD, progressive disease.

*Censored data. Dogs considered long-term survivors (with an OS >4 months) are underlined.
